# Comparative Assessment of NMR Probes for the Experimental Description of Protein Folding Pathways with High-Pressure NMR

**DOI:** 10.3390/biology10070656

**Published:** 2021-07-12

**Authors:** Vincent Van Deuren, Yin-Shan Yang, Karine de Guillen, Cécile Dubois, Catherine Anne Royer, Christian Roumestand, Philippe Barthe

**Affiliations:** 1 Centre de Biologie Structurale, CNRS, INSERM, Université de Montpellier, 34090 Montpellier, France; vincent.vandeuren@kuleuven.be (V.V.D.); yang@cbs.cnrs.fr (Y.-S.Y.); karine@cbs.cnrs.fr (K.d.G.); cecile.dubois@cbs.cnrs.fr (C.D.); 2 Department of Biological Sciences, Rensselaer Polytechnic Institute, Troy, NY 12180, USA; royerc@rpi.edu

**Keywords:** protein folding, NMR, high hydrostatic pressure, thermodynamic stability

## Abstract

**Simple Summary:**

During the last decade, high-pressure multidimensional NMR has emerged as a very powerful tool to describe the folding landscapes of proteins. This is (i) because pressure is a gentle perturbation, the effects of which originate from local properties of the folded state, contrary to chemical or thermal denaturation, and (ii) because multidimensional NMR intrinsically provides multiple probes strategically scattered on the three-dimensional structure of the protein, allowing a quasi-atomic resolution to describe the folding pathway. Residue-specific information obtained from these probes can be used to describe protein folding pathways through the calculation of NMR-derived fractional probabilities of contact at increasing pressure. Here, we used this strategy to evaluate and compare the results obtained from NH amide, CαHα, and CH_3_ groups when used as NMR probes to explore the folding pathway of the model protein ∆+PHS Staphylococcal Nuclease.

**Abstract:**

Multidimensional NMR intrinsically provides multiple probes that can be used for deciphering the folding pathways of proteins: NH amide and CαHα groups are strategically located on the backbone of the protein, while CH_3_ groups, on the side-chain of methylated residues, are involved in important stabilizing interactions in the hydrophobic core. Combined with high hydrostatic pressure, these observables provide a powerful tool to explore the conformational landscapes of proteins. In the present study, we made a comparative assessment of the NH, CαHα, and CH_3_ groups for analyzing the unfolding pathway of ∆+PHS Staphylococcal Nuclease. These probes yield a similar description of the folding pathway, with virtually identical thermodynamic parameters for the unfolding reaction, despite some notable differences. Thus, if partial unfolding begins at identical pressure for these observables (especially in the case of backbone probes) and concerns similar regions of the molecule, the residues involved in contact losses are not necessarily the same. In addition, an unexpected slight shift toward higher pressure was observed in the sequence of the scenario of unfolding with CαHα when compared to amide groups.

## 1. Introduction

Hydrostatic pressure is a method of choice in studies of protein folding. This “relatively gentle” perturbation is generally reversible and provides access to thermodynamic parameters at atmospheric pressure, characteristic of the folding/unfolding reaction [1,2,3,4]. Contrary to chemical denaturation, it does not modify the composition of the system. Additionally, it does not modify the charges of the molecule, contrary to pH denaturation. Finally, the ∆*C_p_* of the system remains constant over the full reaction, contrary to thermal denaturation. Following the Le Châtelier principle, pressure leads to the unfolding of protein because the molar volume of the unfolded state is smaller than that of the folded state, i.e., the volume change upon unfolding $\Delta V_{u}^{0}$ is negative [5,6]. It is now largely admitted that the elimination of the solvent-excluded internal voids due to imperfect protein packing likely represents the largest contribution to the magnitude of $\Delta V_{u}^{0}$ [7,8,9,10,11]. Because the distribution of solvent-excluded cavities is specific to each protein’s structure, pressure-induced unfolding originates from local properties of the folded state, contrary to unfolding by temperature or chemical denaturants, the effects of which depend on the amount of exposed surface area in the unfolded state.

Due to recent methodological and technical advances, high-pressure NMR (HP-NMR) spectroscopy has emerged as a particularly powerful tool to obtain high-resolution structural description of the protein folding landscape (for reviews [12,13,14,15,16,17,18]). This is primarily because an abundance of site-specific probes can be studied simultaneously in a multidimensional NMR spectrum. Strategically located on the protein backbone, amide group resonances (^1^H,^15^N) are currently used to monitor the unfolding reaction: each amino acid bears a HN group, with the exception of proline, and is well resolved in the proton and nitrogen-15 spectral dimensions of heteronuclear 2D [^1^H-^15^N]-HSQC experiments. Hence, they represent good observables to describe the protein folding pathway at a residue-level resolution. On the other hand, amide protons are acidic and prone to exchange with water at a rate that depends on pressure, thus introducing possible bias in the measurement of thermodynamics parameters $\Delta V_{u}^{0}$, $\Delta G_{u}^{0}$). CαHα group resonances (^1^H,^13^C) could represent an alternative to probe protein unfolding with variable-pressure 2D [^1^H-^13^C]-HSQC experiments. They are also ideally distributed along the protein backbone, but Hα protons are not exchangeable, contrary to amide protons. Finally, CH_3_ group NMR resonances can also serve probe protein unfolding. Residues bearing methylated side-chains (A, T, M, V, I, L) are usually well distributed in the structure of globular soluble proteins and provide decisive stabilizing hydrophobic interactions in the core of the molecule. Far fewer than amide or CαHα groups, the strategic location of CH_3_ groups can nevertheless yield important information on the loss of tertiary contacts during protein unfolding. In the present study, we have used HP-NMR spectroscopy to explore the folding pathway of Staphylococcal Nuclease (SNase) and compare the description given by different NMR probes: amide groups, CαHα groups, and CH_3_ groups.

**Figure 1:**
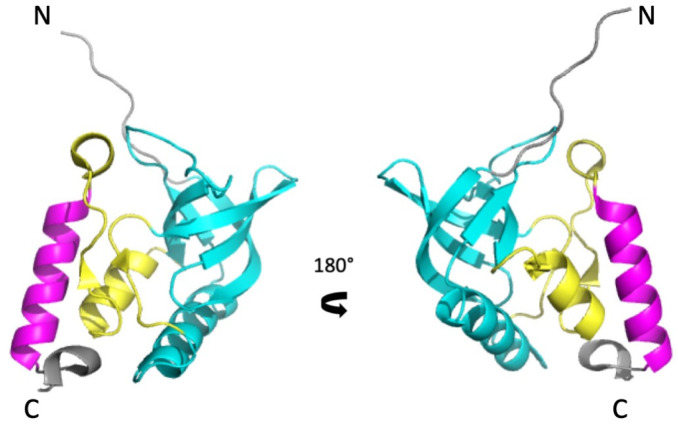
The subdomain organization of the structure of Staphylococcal Nuclease (PDB ID: 3LX0)**.** Two views (180° rotation along the vertical axis) of a cartoon representation of the 3D structure of ∆+PHS Staphylococcal Nuclease. SubD1, IntD, and SubD2 subdomains are colored in cyan, yellow, and magenta, respectively.

The model protein used in this study (SNase) is a small extracellular enzyme (149 residues) produced by *Staphylococcus aureus* that degrades both DNA and RNA to short oligonucleotides in the presence of Ca^2+^. It belongs to the OB-fold (Oligonucleotide/oligosaccharide-Binding) superfamily, a widely represented architecture characterized by a 5-stranded β-barrel, capped by an α-helix found between strands 3 and 4 [19,20]. It has long served as model system for protein folding studies [21,22]. In spite of its moderate complexity, SNase displays three structural subdomains (Figure 1) [23,24,25]. The major N-terminal subdomain (SubD1) consists of the OB-fold itself, encompassing the first 96 residues. The C-terminal helix (residues 122–134) forms the second subdomain (SubD2), linked to SubD1 by an interfacial domain (IntD), essentially formed by a short helix (residues 99–105), a mini-ß-sheet (residues 39–40 and 110–111), and loops. The C-terminal end of the molecule forms a turn (residues 137–141), bearing the sole tryptophan residue (W140), which stabilizes the protein via multiple contacts [26,27]. This organization has been confirmed for a SNase mutant by H/D exchange experiments that reveal three foldons, which correspond more or less to the subdomain description [28]. 

As in previous published studies, we used a hyper-stable variant of SNase known as ∆+PHS SNase (∆∆*G* ≈ 7 kcal/mol when compared to the wild-type protein) [9,10,29]. This variant bears stabilizing substitutions (G50F, V51N, P117G, H124L, and S128A) and a deletion of the mobile Ω loop (residues 44–49), which is part of the active site of the enzyme.

## 2. Materials and Methods

### 2.1. Protein Expression and Purification

The highly stable ∆+PHS form of SNase was expressed and purified, as described in detail by Shortle and Meeker [30]. The construct ∆+PHS SNase was sub-cloned in pET24a plasmids with kanamycin resistance and introduced in BL21(DE3) bacteria using the heat-shock method. Uniform ^15^N/^13^C labeling was obtained by growing cells in minimal M9 medium containing ^15^NH4Cl/^13^C-u-labeled glucose as the sole nitrogen and carbon sources. Protein was expressed overnight at 20 °C after induction with 0.2 mM IPTG. The cells were then centrifuged at 5000 rpm for 10 min, and the bacterial pellet was resuspended in an ice-cold buffer containing 25 mM Tris pH8 buffer 2.5 mM EDTA and 6 M Urea (buffer#1). This suspension was kept at 4 °C for 20 min under shaking condition and then centrifugated at 8000 rpm for 15 min. The pellet was resuspended in the same cold buffer but containing 400 mM NaCl (buffer#2). This new suspension was kept at 4 °C for 30 min under gentle shaking condition and then centrifugated at 8000 rpm for 15 min. An equal volume of ice-cold 100% ethanol was added to the supernatant, and precipitation was promoted for 3h at −20 °C. The precipitate was discarded after centrifugation (20 min at 8000 rpm), and an additional two-volumes of ice-cold ethanol was added to the supernatant, and the solution was kept overnight at −20 °C. After the last centrifugation (20 min at 8000 rpm), the precipitate was solubilized in 30 mL of buffer#1 and injected in a Sephadex (Merck-Sigma-Aldrich, France) cationic resin column. Elution was done with a linear gradient of buffer#2. The fractions containing the pure protein were pooled, and the sample was first dialyzed in Tris buffer (10 mM, pH 7) containing 1 M KCl overnight, with stirring under refrigeration, and subsequently in Tris 10 mM pH 7 for 2 h. For NMR studies, the solution was concentrated to about 1 mM (protein concentration), aliquoted to 0.3 mL samples, and freeze-dried. Yields of purified protein were on the order of 60 mg/L. Protein concentration was determined at 280 nm using an extinction coefficient of 0.93.

### 2.2. Protein NMR Resonance Assignment

The assignment of the amide group ^1^H and ^15^N resonances have been reported in previous works [6,7,21]. The assignment of the CαHα group and CH_3_ group ^1^H and ^13^C resonances were achieved through a 3D [^1^H,^13^C] TOCSY-HSQC (isotropic mixing: 60 ms) NMR experiment performed on a 1 mM ^15^N,^13^C-labeled ∆+PHS sample (0.2 mL in a conventional 3 mm glass tube), dissolved in Tris 10 mM deuterated buffer at pH 7 (uncorrected from isotopic effects). Additionally, a [^1^H,^13^C] NOESY-HSQC (mixing time: 200 ms) was recorded, which helped us solve some ambiguities. Experiments were recorded at 20 °C on a Bruker AVANCE III (Bruker Biospin, Wissenbourg, France) 700 MHz equipped with a 5 mm Z-gradient TCI cryogenic probe head. ^1^H chemical shifts were directly referenced to the methyl resonance of DSS, while ^13^C and ^15^N chemical shifts were referenced indirectly to the ^13^C/^1^H and ^15^N/^1^H absolute frequency ratios. All NMR experiments were processed with Gifa [31].

### 2.3. Protein Unfolding

[^1^H,^15^N] and [^1^H,^13^C] HSQC experiments were recorded at 20 °C on a Bruker AVANCE III 600 MHz spectrometer, equipped with a TXI probe operating at ambient, and at 15 different hydrostatic pressures (1, 30, 200, 400, 600, 800, 1000, 1200, 1400, 1600, 1800, 2000, 2200, 2400, and 2500 bar). The doubly-labeled ^15^N,^13^C protein sample was dissolved at a concentration of 1 mM in a Tris 10 mM pH 7 aqueous buffer (+10% D_2_O for the lock) and used with a 5 mm o.d. ceramic tube (0.33 mL of sample volume) from Daedalus Innovations (Aston, PA, USA). Guanidinium chloride was added to the sample (1.8 M) in order to drag the protein stability into the pressure range allowed by the experimental set-up (1–2500 bar). Hydrostatic pressure was applied to the sample directly within the magnet using the Xtreme Syringe Pump, also from Daedalus Innovations. Pressure was transmitted by mineral oil, so that no physical separation was needed between the aqueous buffer containing the protein and the transmitting fluid. Each pressure jump was followed by a 12-h relaxation time to allow the protein to reach a steady-state equilibrium before running the 2D experiments. 2D [^1^H,^15^N] and [^1^H,^13^C] HSQC were recorded sequentially, hence on the same sample and strictly in the same experimental conditions. These experiments were recorded using gradient coherence selection through pulsed field gradients, yielding an excellent suppression of the water resonance in the proton dimension.

The intensities of cross peaks corresponding to NH, CαHα, or CH_3_ groups were measured for the folded species at each pressure, and their decrease in pressure was then fitted with a two-state model:

(1) $$I=\frac{I_{f}+I_{u}e^{-\left(\Delta G_{u}^{0}+p\Delta V_{u}^{0}\right)/RT}}{1+\;e^{-\left(\Delta G_{u}^{0}+p\Delta V_{u}^{0}\right)/RT}}$$

where *I* is the intensity of a native state cross peak measured at a given pressure and *I_f_* and *I_u_* correspond to the cross peak intensities in the folded state (1 bar, *I_f_* = *I_max_*) and in the unfolded state (2500 bar, *I_u_* = *I_min_*), respectively. $\Delta G_{u}^{0}$ stands for the residue specific apparent free energy of unfolding at atmospheric pressure. $\Delta V_{u}^{0}$ corresponds to the residue specific apparent volume of unfolding for pressure denaturation.

Native contact maps were obtained by using software CMView [http://www.bioinformatics.org/cmview/] (accessed on 25 March 2020) with a threshold of 8.5 Å around the Cα of each residue, using the crystal structure of ∆+PHS SNase (PDB ID: 3LX0).

## 3. Results

### 3.1. NMR Resonance Assignment

**Figure 2:**
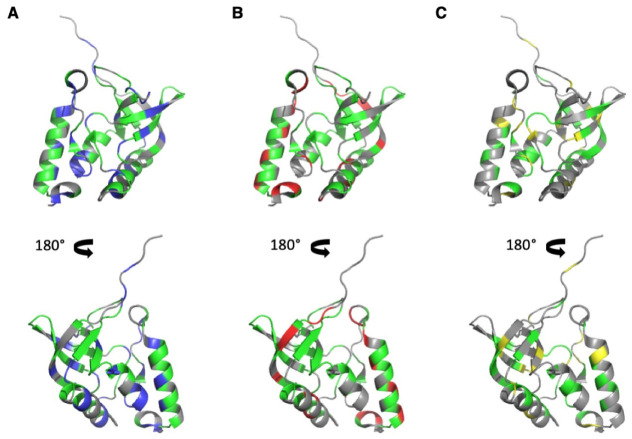
Distribution of the NMR probes used for the HP-NMR denaturation study on the 3D structure of ∆+PHS SNase (PDB ID: 3LX0). (**A**–**C**): Two views (180° rotation along the vertical axis) of a cartoon representation of the 3D structure of ∆+PHS Staphylococcal Nuclease. In (**A**,**B**), the colored residues correspond to the NH (**A**) (in blue and green) and CαHα (**B**) (in red and green) groups, which were assigned and gave neither overlapping cross peaks in the folded state nor in between the folded and unfolded states at 20 °C in the corresponding HSQC spectra. The residues colored in green correspond to residues where both the NH and the CαHα groups gave no overlapping cross peaks. In (**C**), the colored residues (in yellow and green) correspond to all methylated residues. Those colored in green correspond to residues that gave no overlapping in the 2D [^1^H,^13^C] HSQC spectra (folded and unfolded states). In this figure, all residues colored in green were used for the HP NMR study.

Amide resonances (^1^H and ^15^N) were assigned for all amide groups in previous work [9,10,30], and those of CαHα and CH_3_ groups (^1^H and ^13^C) were assigned through 3D ^13^C-edited TOCSY and NOESY experiments recorded on a ^15^N,^13^C-uniformly enriched ∆+PHS SNase sample dissolved in a deuterated buffer (see Materials and Methods). The assigned HSQC 2D spectra corresponding to these sets of resonances are given as Supplementary Materials (Figures S1–S3). A total of 100 amide cross peaks (73% of the residues) and 81 CαHα cross peaks (59% of the residues) gave neither overlapping cross peaks in the folded state nor in between the folded and unfolded states at 20 °C. Nevertheless, for the accuracy of the comparison, we considered only residues where both NH and CαHα groups were resolvable for further analysis (61 residues, 45% of the sequence). Our construct contains 51 methylated residues (37% of the sequence): 14 alanine, 8 threonine, 4 methionine, 8 valine, 12 leucine, and 5 isoleucine residues. While all CH_3_ groups can be assigned unambiguously, only 33 (24% of the sequence) gave cross peaks (at least one cross peak for residues I, L, V, bearing two methyl groups), which showed no overlap in both the folded state or between the folded and unfolded states at 20 °C, and were considered for further analysis. The distribution of the selected residues on the 3D structure of SNase is displayed in Figure 2.

### 3.2. HP-NMR Denaturation Study: Measuring the Thermodynamic Parameters for the Unfolding Reaction

**Figure 3:**
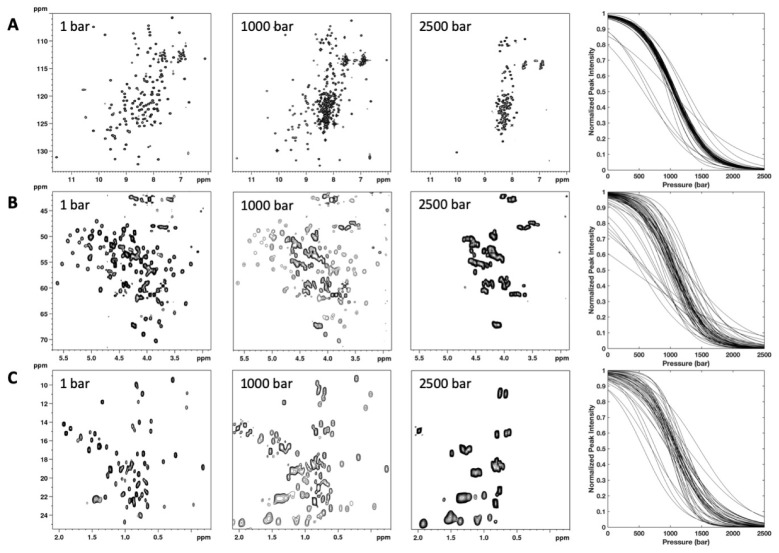
NMR detected high-pressure unfolding of ∆+PHS SNase. From left to right, examples of 2D [^1^H,^15^N] (**A**) and [^1^H,^13^C] (**B**,**C**) HSQC spectra recorded at 1, 1000, and 2500 bar are displayed. In (**B**,**C**), only a zoom on the CαHα or on the CH_3_ cross peak region, respectively, is displayed. The rightmost panels report overlays of the normalized residue-specific denaturation curves obtained from the fits of the pressure-dependent sigmoidal decrease of (**A**) NH amide, (**B**) CαHα, and **C**) CH_3_ cross peak intensities in the corresponding HSQC spectra with Equation [1]. Representative examples of experimental fits are given in Supplementary Materials (Figure S4).

2D [^1^H,^15^N] and [^1^H,^13^C] HSQC experiments were recorded at variable pressure within the 1–2500 bar pressure range and at 20 °C. In all these spectra, the intensity of each native state cross peak decreases as a function of pressure, while the intensity of peaks corresponding to the unfolded state increases concomitantly (Figure 3). This supports a slow equilibrium on the NMR timescale for each residue between the native and unfolded state and a two-state transition for each residue between their native/unfolded states during the unfolding process. Thus, this simple model can be used to interpret the loss of intensity for each native state cross peak, even though the global protein unfolding does not likely conform to a two-state transition, locally [18]. 

**Figure 4:**
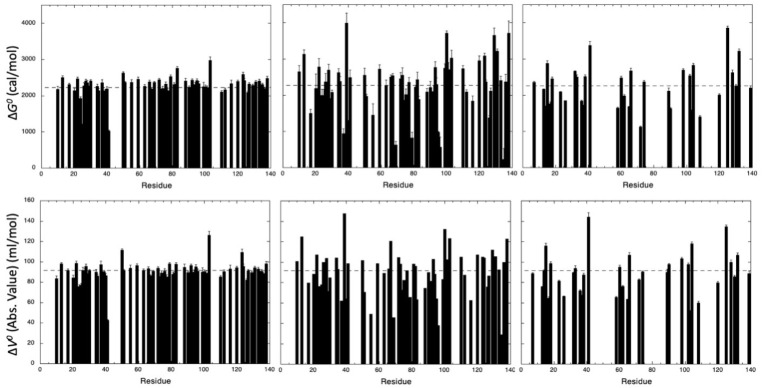
Thermodynamic parameters measured for the unfolding reaction of ∆+PHS SNase. ∆*G*^0^ (upper panels) and ∆*V*^0^ (absolute value, lower panels) obtained from the fit with Equation (1) of the pressure-dependent sigmoidal decrease of the residue cross peak intensities of (from left to right) NH amide groups, CαHα groups, and CH_3_ groups. For I, L and V residues, when denaturation curves have been obtained for each of the two cross peaks (corresponding to the two methyl groups), averaged values of ∆*G*^0^ and ∆*V*^0^ are displayed. The dashed lines represent the mean values of the measured thermodynamic parameters. The values of ∆*G*^0^, ∆*V*^0^ and also P_1/2_ (the half-denaturation pressure) are gathered in Table S1 (Supplementary Materials).

A substantial number of local NMR probes (61 NH and CαHα cross peaks, 33 CH_3_ cross peaks) can be accurately fitted to the two-state pressure-induced unfolding model described in the Materials and Methods (Equation (1)), yielding local apparent values for $\Delta G_{u}^{0}$ and $\Delta V_{u}^{0}$ (Figure 4). Although the two-state model was adequate to fit all individual unfolding curves at a residue level, significantly different residue-specific values for apparent free energy $\Delta G_{u}^{0}$ and apparent volume change $\Delta V_{u}^{0}$ of unfolding were observed, suggesting a deviation from a two-state behavior for the global unfolding of the protein for all probes used for the study.

Interestingly, although values obtained from CαHα groups and CH_3_ groups show a broader distribution than those obtained from NH groups, virtually identical average values (within the experimental errors) were obtained for the three probes used. Amide groups yield an average $\Delta G_{u}^{0}$ value of 2282 ± 275 cal/mol and a $\Delta V_{u}^{0}$ of −91 ± 10 mL/mol, CαHα groups yield an average $\Delta G_{u}^{0}$ value of 2245 ± 763 cal/mol and a $\Delta V_{u}^{0}$ of −92 ± 22 mL/mol, and CH_3_ groups yield an average $\Delta G_{u}^{0}$ value of 2252 ± 597 cal/mol and a $\Delta V_{u}^{0}$ of −91 ± 21 mL/mol (Figure 4). Note that, for NH and CαHα probes, these values were not biased by the residue selection that we made, keeping only those for which residue-specific curves could be obtained for both atom groups. When considering the 100 amide cross peaks and the 81 CαHα groups that can be fitted accurately with Equation (1) (Supplementary Materials, Figure S5), similar values were obtained: $\Delta G_{u}^{0}$ value of 2221 ± 391 cal/mol and a $\Delta V_{u}^{0}$ of −90 ± 13 mL/mol for amide cross peaks; $\Delta G_{u}^{0}$ value of 2224 ± 721 cal/mol and a $\Delta V_{u}^{0}$ of −91 ± 22 mL/mol for CαHα cross peaks. It should be noticed that the apparent global values obtained for $\Delta G_{u}^{0}$ and $\Delta V_{u}^{0}$ (2394 ± 82 cal/mol and −87 ± 3 mL/mol, respectively) were also in good agreement with the average values of the corresponding apparent residue-specific values extracted from NH, CαHα, or CH_3_ denaturation curves, supporting the two-state equilibrium regime for folding or unfolding. These global values were obtained by fitting the increase of the resonance at 0.8 ppm (which corresponds roughly to the resonance of the methyl groups in the unfolded state) on a series of 1D spectra recorded at increasing pressure [18] (Supplementary Materials, Figure S6). This constitutes reassuring results, since the global (or averaged) thermodynamic parameters measured by the different NMR probes are supposed to reflect a similar global behavior of the same system under the same perturbation, even though differences can be expected at a local, residue-specific level.

### 3.3. HP-NMR Denaturation Study: Exploring the Folding Pathways of ∆+PHS SNase

**Figure 5:**
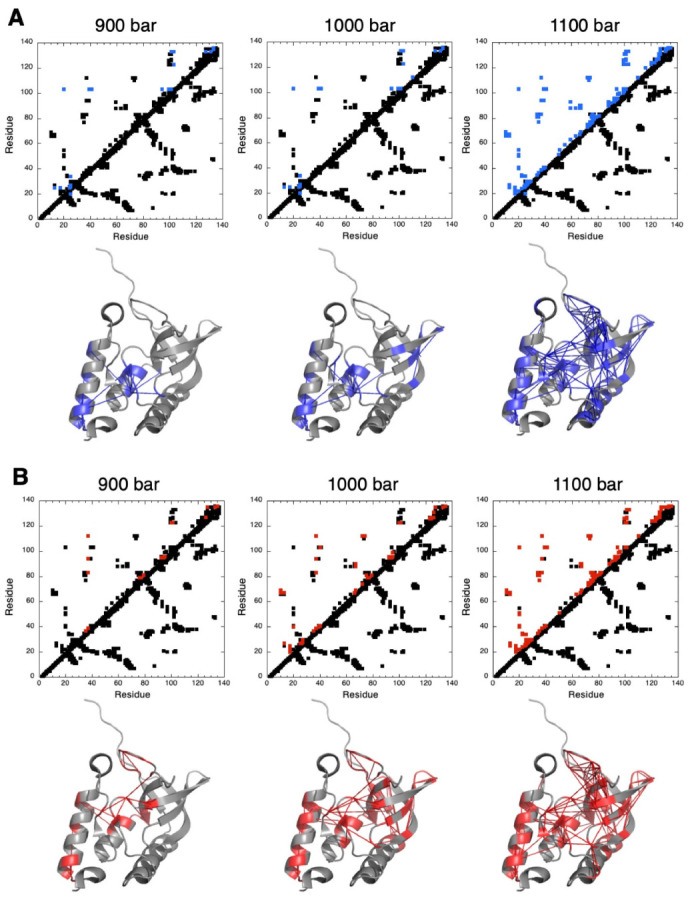
Pressure denaturation of ∆+PHS SNase probed by NH and CαHα backbone atoms. (**A**) Upper panels: contact maps built from the crystal structure of ∆+PHS SNase (PDB ID: 3LX0) at 900, 1000, and 1100 bar, as indicated. Contacts below the diagonal have been calculated with CMview: they correspond to residues where the distance to the corresponding Cα is lower than 8.5 Å. Above the diagonal, the contacts displayed correspond to residues for which fractional probability can be measured from normalized residue-specific denaturation curves obtained from NH cross peaks. In addition, contacts have been colored in blue when contact probabilities *P_ij_* lower than 0.5 are observed. Lower panels: visualization of the probabilities of contact on ribbon representations of ∆+PHS SNase at 900, 1000, and 1100 bar, as indicated. The blue lines represent contacts that are significantly weakened (*P_ij_* ≤ 0.5) at the indicated pressure. Residues involved in these contacts are also colored in blue. (**B**) Similar as in (**A**), but the fractional probabilities have been calculated from normalized residue-specific denaturation curves obtained from CαHα cross peaks. In the contact maps, contacts have been colored in red when contact probabilities P_ij_ are lower than 0.5. These weakened contacts are visualized on the 3D structure of ∆+PHS SNase by red lines, and the residues involved in these contacts are also colored in red.

In the case of NH and CαHα groups, we used the now classical strategy to track possible intermediates in the folding pathway of ∆+PHS SNase [10,18]. This strategy is based on exploiting the information brought by normalized residue-specific denaturation curves: for a given residue “*i*”, the value of 1 at a given pressure (*I* = *I_f_* = 1; Equation (1)) can be associated with a probability $P_{i}$ of 1 (100%) to find this residue “*i*” in the native state, while for a residue “*j*”, the value of 0 at the same pressure, (*I* = *I_U_* = 0) can be associated with a probability $P_{j}$ equal to zero to find this residue “*j*” in a native state. These probabilities are called fractional probabilities because they are related to the “native fraction” for a given residue. If these two residues *i* and *j* are in an intermediate situation (0 < $P_{i}$ and $P_{j}$ < 1) at a given pressure, and if they are in contact in the native 3D structure (at atmospheric pressure), their fractional probability *P_ij_*, in contact at this pressure, is given by the geometric mean of the two individual probabilities: *P_ij_* = $\sqrt{P_{i}\times P_{j}}$ [12] (Figure 5) [18,32].

Whichever the probe considered (NH or CαHα groups), unfolding appears to follow a similar scenario. At 900 bar, we observed a partial loss of contacts (*P_ij_* ≤ 0.5) between residues from the interfacial domain (IntD) to residues in SubD1 (the OB-fold domain) and SubD2 (the C-terminal helix) (Figure 5). Although this partial unfolding globally concerns the same area of the 3D structure whatever the probe used, in the details, the residues directly involved are not the same. When using amide groups, fractional contact probabilities below 0.5 were measured essentially for two central residues: L103, located in the short helix of IntD, and K133, located in SubD2. Residue L103 displays weakened contacts with residue G20 in SubD1, with residues V39, D40, T41, A94, V99, N100, and E101 within IntD itself and with residues Q123 and K133, located in SubD2. Residue K133 displays weakened contacts with residues in SubD2 (K127, Q131, E135, and K136) and with residues in IntD (E101 and L103). When considering CαHα groups, weakened contacts essentially concern residue L37 in IntD (with V39 and D83 in subD1 and R35, A94, and A112 within IntD itself), residue G79, located in a loop connecting the β4-strand to the β5-strand from the β-barrel of SubD1 (with F76, D77, and R81 in the same loop), residue Q123 in SubD2 (with N100 and E101 in IntD), and residue E135, also in SubD2 (with K127 and K133 in SubD2) (Figure 5). 

At 1000 bar, partial unfolding concerns additional residues in the same areas and the loss of some contacts in the OB-fold itself between residues in the C-terminal turn of the helix and in the first ß-strand of the barrel. Intriguingly, at this pressure, partial unfolding seems to concern more residues when considering fractional probabilities calculated from residue-specific denaturation curves obtained from CαHα probes than from those obtained from NH amide probes. More weakened contacts (*P_ij_* ≤ 0.5) can be detected in the fractional contact maps built from CαHα residue-specific denaturation curves (involving a total of 47 residues) than from those from NH residue-specific denaturation curves (involving only 24 residues). This suggests a sharper unfolding transition when probed by the CαHα groups.

Indeed, at a slightly higher pressure (1050 bar), the number of weakened contacts detected from NH probes becomes closer (38 residues involved) to that detected from CαHα probes at 1000 bar (Supplementary Materials, Figure S7). Moreover, among these 38 residues, 31 (≈82%) also belong to the group of 47 residues involved in weakened contacts (*P_ij_* ≤ 0.5) when probing the unfolding transition with CαHα groups (at 1000 bar). This strongly suggests a similar scenario for the partial unfolding of ∆+PHS SNase, which is slightly shifted to a higher pressure when described by NH probes.

Finally, at 1100 bar, regardless of the probe under consideration, a quasi-global unfolding of the molecule is observed, with the loss (*P_ij_* ≤ 0.5) of most of the contacts in the 3D structure, including those stabilizing the ß-barrel in the OB-fold domain (Figure 5).

**Figure 6:**
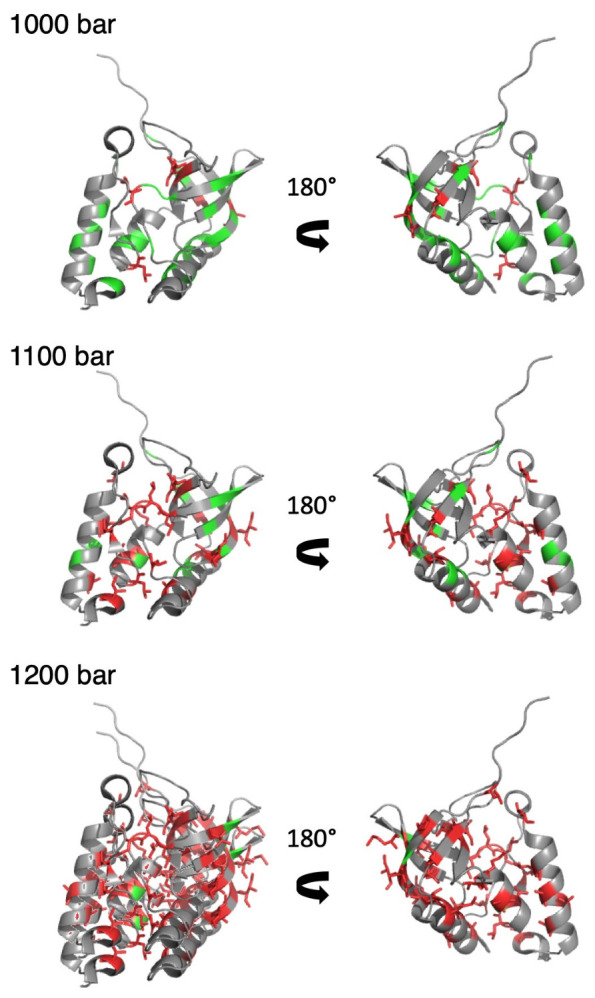
Pressure denaturation of ∆+PHS SNase probed by side chain CH_3_ groups. Two views related by a 180° rotation along the vertical axis of a ribbon representation of the 3D structure of ∆+PHS SNase (PDB ID: 3LX0). The green and red color correspond to methylated residues, with fractional probabilities greater or lower than 0.5, respectively. In addition, the side chains of the methylated residues displaying fractional probabilities lower than 0.5 are represented by red sticks. Results obtained at three different pressures (1000, 1100, and 1200 bar) are displayed from top to bottom, as indicated.

When compared to NH or CαHα groups, methyl groups constitute scarce probes for analyzing the folding landscape of the protein (33 selected CH_3_ groups against 61 for NH or CαHα, in the results presented here), and contacts between them are few. Thus, we directly used the individual fractional probabilities *P_i_* instead of deriving fractional probabilities of contact *P_ij_* between residues. Using this simplified approach, it was not possible to determine which contacts in the 3D structure were lost, but we assessed whether a methylated residue was in a native or unfolded environment at a given pressure. The first methylated residues exhibiting fractional probabilities lower than 0.5 were detected at 1000 bar. When displayed in the 3D structure of ∆+PHS SNase, they were located either in the interfacial domain or in the OB-fold domain and C-terminal helix, with side chains pointing toward the interfacial domain (Figure 6). Also consisted was L14, a residue located on the first ß-strand of the ß-barrel, the side chain of which pointing toward the C-terminal turn of the helix of the OB-fold domain. Fractional probabilities lower than 0.5 concern only residues located in these two areas up to a pressure of 1100 bar. Above (1200 bar), most of the methylated residues report an unfolded environment. Thus, it seems that the folding pathway probed by the methylated side chains is very similar to that probed by the backbone atoms.

## 4. Discussion

Due to the better resolution of the 2D [^1^H,^15^N] HSQC spectrum, NH amide cross peaks provide the most important number of residue-specific NMR probes to explore the folding landscape of ∆+PHS SNase. Indeed, 100 residue-specific denaturation curves can be obtained from non-overlapping cross peaks over 133 non-proline residues. Due to more severe overlapping cross peaks either in the folded state 2D [^1^H,^13^C] HSQC spectrum or between the unfolded and unfolded state spectra, a more limited number (81 over 137 residues) can be obtained from CαHα cross peaks, and this number drops to 33 (over 51 methylated residues) when considering the CH_3_ cross peaks. In addition, some CαHα cross peaks can be obscured possibly by the water resonance, in the middle of the Hα proton resonances, even if, in the present case, the water suppression scheme (coherence selection through pulsed field gradients) worked well, yielding virtually no artefact, probably due to the rather high protein concentration (1 mM) in the NMR sample. 

Whichever the probes used, the fit of cross peak intensity decrease with pressure to a two-state model yields virtually identical average values for the apparent thermodynamic parameters $\Delta G_{u}^{0}$ and $\Delta V_{u}^{0}$, supporting the theory that NH, CαHα, or CH_3_ probes equally sense the denaturation reaction. In the detail, we observed a broader distribution of the residue-specific values of $\Delta G_{u}^{0}$ and $\Delta V_{u}^{0}$ in the case of CαHα and CH_3_ groups than in the case of NH groups. Thus, apparently, the unfolding reaction seems more cooperative when probed with NH amide groups. We do not have a clear explanation for this observation, except maybe the fact that all the amide groups are involved in H-bonds, contrary to CαHα or CH_3_ groups. Breaking these H-bonds is mandatory for a NH groups to sense a disordered environment. Since the free-energy associated with H-bonds does not vary significantly within the protein sequence, this probably confers a common and quasi-identical “unfolding contribution” for all amide groups. The differences observed between the apparent thermodynamic parameters $\Delta G_{u}^{0}$ and $\Delta V_{u}^{0}$, measured for NH groups, should be due to the contribution of the variable distribution of water-excluded voids around them, yielding significant differences in their pressure sensitivity. Since CαHα and CH_3_ groups are not involved in H-bonds, they should be sensitive only to this latter contribution. 

Fractional probabilities of contact *P_ij_*, deduced from either NH or CαHα NMR probes, globally describe a similar folding pathway: the protein denaturation starts at 900 bar with the partial unfolding of IntD and SubD2 sub-domains, whereas SubD1 (the OB-fold domain) remains virtually non-affected until 1100 bar. These results are consistent with those obtained in previous work, demonstrating the existence of a folding intermediate state, where only the OB-fold domain is folded [10]. A similar folding pathway can also be observed when using CH_3_ probes. When increasing pressure, the first methylated residues sensing an unfolded environment are located in IntD or in SubD1 and SubD2 but with side chains pointing toward IntD. This supports local unfolding of IntD and SubD2 sub-domains. Nevertheless, since fractional probabilities of contact cannot be calculated from CH_3_ probes, a structural description of a possible folding intermediate becomes difficult. Interestingly, the global value for $\Delta G_{u}^{0}$ and $\Delta V_{u}^{0}$ measured on 1D spectra from the increase with pressure of the resonance corresponding to the unfolded CH_3_ groups is similar to the average values of residue-specific $\Delta G_{u}^{0}$ and $\Delta V_{u}^{0}$ measured on 2D spectra for each individual CH_3_ group. This means that the thermodynamic parameters measured at equilibrium for ∆+PHS SNase are similar for the folding and unfolding reaction. This strongly supports a two-state equilibrium between the folded and unfolded states, without the appearance of a pressure-stabilized folding intermediate (or molten-globule), as has been observed for the pp32 L60A variant [33]. 

Although residue-specific NH or CαHα probes are located in the backbone of the protein and borne by the same residue, even in cases where local unfolding reported by these probes concerns the same area of the protein, the loss of contacts revealed by their fractional probabilities does not necessarily concern the same residues. As mentioned above, this is probably due to the combined effects of H-bonds (for amide protons) and voids distribution around these atom groups.

Interestingly, the sequence of the scenario of the protein unfolding is slightly shifted to higher pressure when probed with CαHα groups, as if the unfolding transition was sharper when compare to results obtained with NH probes. This is an unexpected result since amide protons are exchangeable. Because solvent exchange increase with pressure [34], this effect should contribute to the intensity decrease of NH cross peaks with pressure. We should observe a steeper slope for the residue-specific denaturation curves obtained from NH amides. The reverse result is obtained, and again we do not have a clear explanation for that. Here also, the involvement of amide protons in H-bonds might be responsible of this rather counter-intuitive effect. On the other hand, this result clearly demonstrates that amide proton exchange with water is negligible and probably contributes marginally to the decrease of amide cross peak intensity in the native state spectrum during unfolding. 

## 5. Conclusions

Among the three NMR probes (NH, CαHα, or CH_3_ groups) studied here to investigate the protein folding pathways, NH and CαHα groups appear to be the best candidates, even if all three of them describe similar scenarios. Indeed, NH and CαHα groups are shared by all (non-proline) residues, although methylated residues constitute only 37% of the ∆+PHS SNase sequence. Moreover, they are strategically located on the protein backbone, an ideal situation to probe protein unfolding. Finally, fractional probabilities of contact can be easily calculated from NH and CαHα probes, which is not the case for CH_3_ probes, yielding a possible structural description of potential folding intermediates [10]. 

Unaffected by water suppression and displaying a better spectral resolution, the 2D [^1^H,^15^N] HSQC spectrum gives a larger number of probes when compared to the CαHα region of the 2D [^1^H,^13^C] HSQC spectrum. Moreover, we have shown here that the contribution of solvent exchange to amide cross peak intensity can be safely neglected, since thermodynamic parameters of the unfolding reaction and the folding pathway described both by NH groups and CαHα groups are similar. In addition, the fact that [^1^H,^15^N] HSQC can be recorded in an unexpansive ^15^N-uniformly-labeled sample (when compared to ^15^N,^13^C-, or even the ^13^C-u-labeled sample) makes NH amide groups very attractive probes for exploring the folding pathway of proteins. On the other hand, due to the more favorable values of natural abundancy and the gyromagnetic ratio of ^13^C, the use of CαHα groups can constitute an interesting alternative for non-recombinant (and non-isotopically-enriched) proteins. Indeed, the use of cryogenic probes allows to record [^1^H,^13^C] HSQC with a good resolution in about 2 h on protein samples of moderate concentration (1–2 mM). In that case, problems due to water suppression can be circumvented by dissolving the protein sample in deuterated buffers.

## Data Availability

Data supporting reported results are available upon request to corresponding authors.

## Supplementary Materials

The following are available online at https://www.mdpi.com/article/10.3390/biology10070656/s1: Table S1: Thermodynamic parameters values obtained for ∆+PHS; Figure S1: Assignment of ∆+PHS SNase amide groups; Figure S2: Assignment of ∆+PHS SNase CαHα groups; Figure S3: Assignment of ∆+PHS SNase CH_3_ groups; Figure S4: Representative examples of experimental fits; Figure S5: Thermodynamic parameters measured for the unfolding reaction of ∆+PHS SNase; Figure S6: Monitoring the unfolding reaction of ∆+PHS SNase with 1D HP-NMR spectroscopy; Figure S7: Fractional contact map obtained at 1050 bar from NH probes.
