# Supplementary material for: Comparative Assessment of NMR Probes for the Experimental Description of Protein Folding Pathways with High-Pressure NMR
Source: Biology (Basel). 2021 Jul 12;10(7):656. doi: 10.3390/biology10070656 (PMC8301334; doi:10.3390/biology10070656)
Supplement: Supplementary file 1 [file biology-10-00656-s001.zip › biology-1274457-supplementary.pdf]

Table S1: Thermodynamic parameters values for ΔG^0, ΔV^0 and $P_{1/2}$ (half-denaturation pressure: $P_{1/2} = \Delta G^0/\Delta V^0$) obtained for Δ+PHS SNase from the fit of residue-specific denaturation curves with equation [1] and for the different NMR probes (CH_3, Cα and NH groups).

Res.	$\Delta G_0(CH_3)$ cal/mol	+/-	$\Delta V(CH_3)$ ml/mol	+/-	$P_{1/2}(CH_3)$ bar	+/-	$\Delta G_0(C_\alpha)$ cal/mol	+/-	$\Delta V(C_\alpha)$ ml/mol	+/-	$P_{1/2}(C_\alpha)$ bar	+/-	$\Delta G_0(NH)$ cal/mol	+/-	$\Delta V(NH)$ ml/mol	+/-	$P_{1/2}(NH)$ bar	+/-
4																		
5																		
8																		
7	2365	15	89	0.6	1115	14												
8																		
9																		
10							2649	168	101	6	1100	139	2182	79	83	3	1096	77
11																		
12																		
13	2165	16	76	0.5	1198	17	3133	127	125	5	1051	85	2503	46	98	2	1070	38
14	1687	22	92	0.9	770	17												
15	2875	73	116	2.7	1041	50												
16																		
17	1772	42	65	1.4	1149	52	1505	105	80	4	792	99	2306	35	91	1	1055	31
18	2457	47	98	1.8	1045	39												
19																		
20							2191	393	88	15	1041	365	2139	69	84	2	1060	65
21																		
22							2785	233	107	9	1089	180	2472	51	99	2	1047	41
23	2090	19	81	0.7	1073	19												
24							1999	165	76	6	1098	180	1924	46	76	2	1062	47
25							1987	178	78	7	1068	188	1188	35	77	1	644	31
26	1847	9	66	0.3	1166	12	2368	230	100	10	992	192	2277	85	92	3	1041	73
27							1962	76	90	3	912	69	2402	57	91	2	1100	51
28							2696	160	104	6	1089	129	2361	57	95	2	1036	49
29							1838	78	72	3	1074	91	2232	38	87	1	1068	34
30							2081	55	85	2	1028	54	2412	27	92	1	1102	24
31																		
32	2664	11	89	0.4	1246	10												
33	2503	66	94	2.4	1115	58												
34							2621	114	104	5	1051	91	2260	61	90	2	1050	55
35							2389	128	93	5	1075	116	2146	52	86	2	1045	49
36	1844	24	72	0.8	1073	26												
37	1680	46	66	1.5	1065	54	946	133	62	5	640	145	2354	91	97	3	1012	75
38	2519	52	87	1.8	1205	49												
39							3991	281	148	11	1131	161	2148	33	90	1	993	28
40							1214	99	64	4	790	113	2196	52	87	2	1061	47
41	3378	105	144	4.2	980	59	2498	198	99	8	1060	165	1032	26	42	1	1016	45
42																		

43																		
44																		
45																		
46																		
47																		
48																		
49																		
50							2548	193	102	8	1048	157	2625	32	111	1	986	23
51							1970	58	71	2	1167	69	2366	34	91	1	1082	30
52																		
53																		
54																		
55							1452	318	49	12	1236	560	2372	77	94	3	1057	65
56																		
57																		
58	1645	23	65	0.8	1052	27												
59							2721	123	98	5	1157	106	2459	55	96	2	1069	46
60	2477	46	95	1.6	1090	39												
61																		
62	1986	39	76	1.3	1094	40												
63							2271	145	89	5	1066	134	2267	46	91	2	1042	41
64																		
65	1680	14	63	0.5	1111	18												
66	2675	69	107	2.6	1050	53	2501	70	93	3	1123	63	2383	53	93	2	1070	47
67							2538	59	120	3	883	40	2190	35	86	1	1062	32
68																		
69							633	89	46	3	581	120	2375	22	91	1	1095	19
70																		
71																		
72	1130	16	83	0.8	571	13	2455	170	104	7	984	136	2445	41	94	2	1091	36
73							2557	206	98	8	1089	180	2129	58	84	2	1065	56
74	2377	29	90	1.0	1100	26	1720	141	73	6	988	158	2203	60	89	2	1037	54
75							2016	163	82	6	1025	162	2118	64	85	2	1043	61
76							1561	144	66	6	996	177	2323	62	91	2	1069	55
77							2354	138	92	6	1076	128	2142	57	85	2	1053	54
78																		
79							819	157	65	6	524	151	2530	45	98	2	1080	36
80																		
81							2233	37	98	2	953	31	2311	53	88	2	1099	48
82							2437	202	96	8	1065	176	2293	33	89	1	1078	30
83							1884	83	63	3	1248	112	2759	46	98	2	1182	39
84																		
85																		
86																		
87																		
88							2094	94	75	3	1175	108	2409	69	95	3	1062	60
89	2118	83	90	3.1	988	73												
90	1636	18	98	0.8	701	13	2209	170	90	7	1027	158	2239	42	89	2	1049	38
91																		
92							2103	174	81	7	1081	178	2427	39	96	1	1054	33

93							2769	155	103	6	1125	126	2076	80	82	3	1056	77
94							2304	174	88	6	1094	162	2307	60	90	2	1076	54
95							981	21	64	1	638	22	2415	58	95	2	1062	50
96							569	279	38	8	629	440	2333	62	91	2	1075	54
97																		
98	2703	35	103	1.3	1097	28												
99	4355	47	163	1.8	1118	24	2744	128	83	4	1384	135	2248	122	90	4	1046	107
100							3718	59	132	2	1177	38	2247	50	90	2	1043	43
101							2716	188	103	8	1108	158	2219	57	89	2	1043	51
102	2540	37	98	1.3	1087	30												
103	1576	21	52	0.6	1273	33	3029	218	123	9	1029	148	2975	98	126	4	987	62
104	2827	52	118	2.0	1004	36												
105																		
106																		
107																		
108	1398	45	60	1.6	979	57												
109																		
110							2732	93	105	4	1091	75	2104	41	85	2	1035	38
111	4525	72	175	2.7	1082	34												
112							2094	66	87	3	1002	62	2165	49	90	2	1004	43
113																		
114																		
115																		
116							1838	148	62	5	1236	204	2331	98	93	4	1049	85
117																		
118																		
119																		
120	2010	38	79	1.4	1058	38	2948	140	107	5	1149	110	2398	40	94	1	1067	34
121																		
122																		
123							3083	73	105	3	1230	60	2584	68	109	3	988	50
124							2373	50	104	2	955	39	2426	54	96	2	1062	45
125	3865	41	135	1.5	1200	26												
126							1376	59	76	3	762	58	2096	35	82	1	1066	35
127							2112	95	87	4	1019	90	2327	32	91	1	1066	28
128	2630	64	100	2.4	1103	54												
129							3649	208	112	6	1366	156	2286	35	90	1	1068	32
130	2265	34	86	1.2	1106	32												
131							3220	62	105	2	1279	50	2380	39	94	1	1059	33
132	3223	59	107	2.0	1264	46												
133							2407	128	93	5	1086	115	2410	41	93	1	1083	35
134																		
135							235	297	29	6	344	506	2310	42	91	1	1064	37
136							2378	196	100	8	997	163	2199	35	89	1	1037	32
137																		
138							3716	331	123	12	1266	232	2484	55	98	2	1059	45
139	2198	68	89	2.5	1037	61												

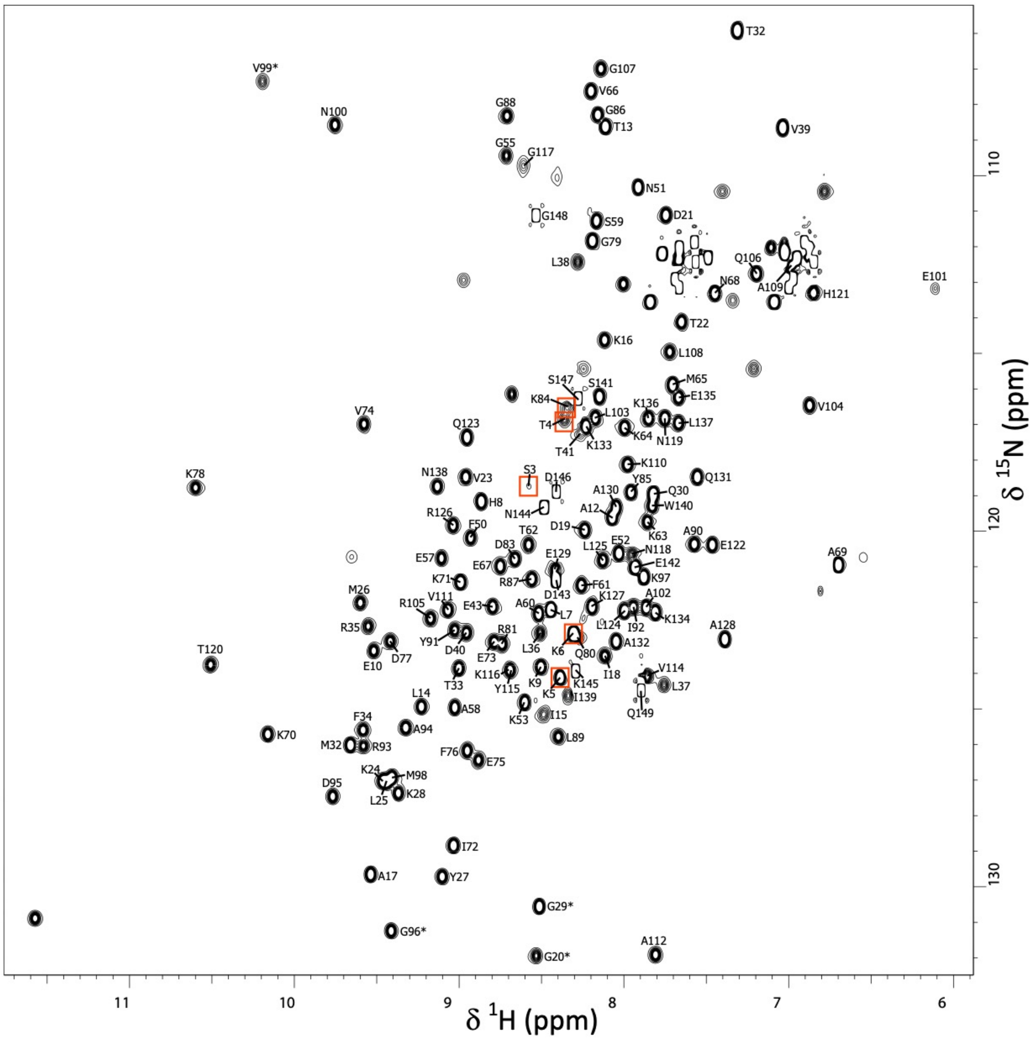

Figure S1: Assignment of Δ+PHS SNase amide groups. [^{1}H-^{15}N] HSQC spectrum of Δ+PHS SNase at 700 MHz, 20 °C on a 1 mM, ^{15}N,^{13}C uniformly labeled sample dissolved in a 10 mM Tris-HCl pH 7.5 (10% D2O for the lock).Amide cross-peaks assignment is indicated using the one-letter amino acid and sequence number code.

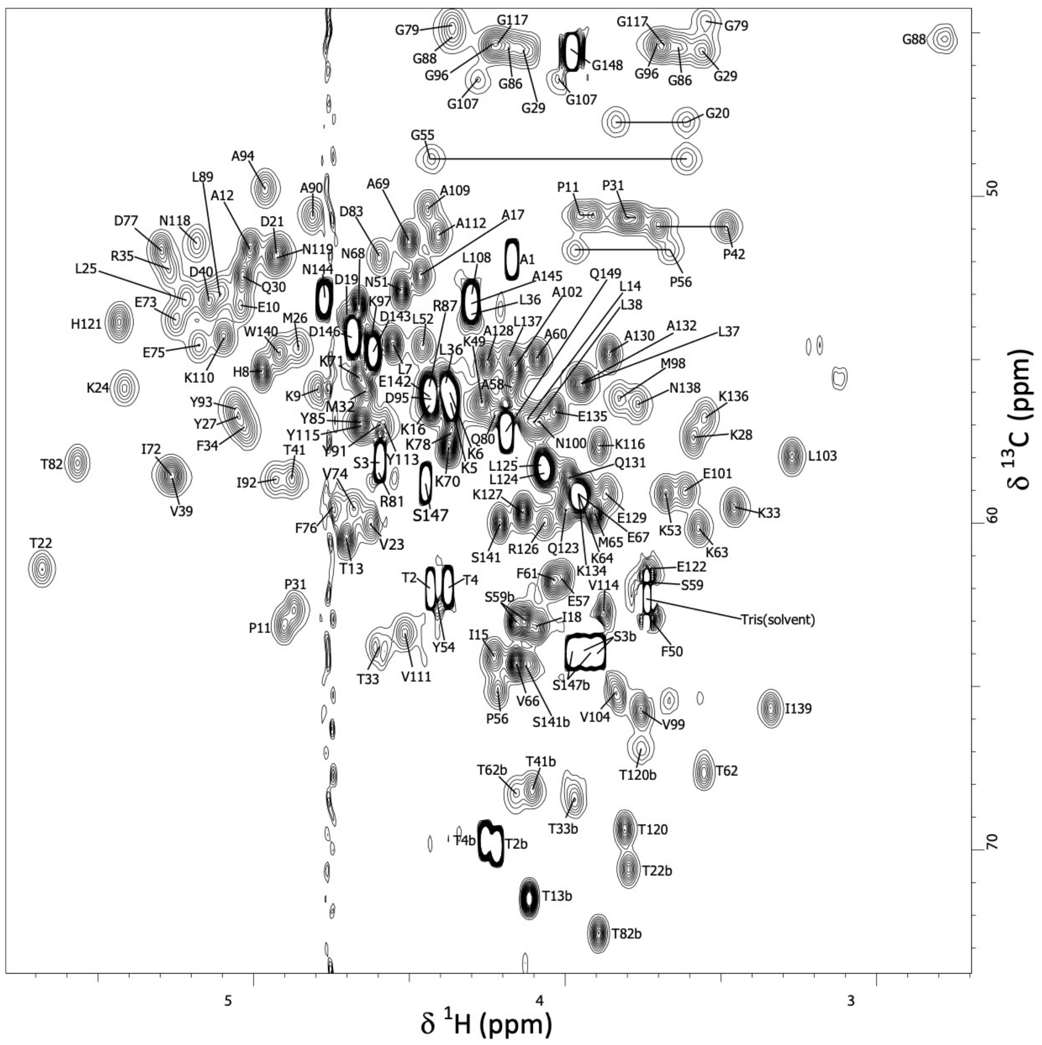

Figure S2: Assignment of Δ+PHS SNase CαHα groups. [^{1}H-^{13}C] HSQC spectrum (zoom on the CαHα cross-peaks region) of Δ+PHS SNase at 700 MHz, 20 °C on a 1 mM, ^{15}N,^{13}C uniformly labeled sample dissolved in a 10 mM Tris-HCl deuterated buffer at pH 7.5. CαHα cross-peaks assignment is indicated using the one-letter amino acid and sequence number code.

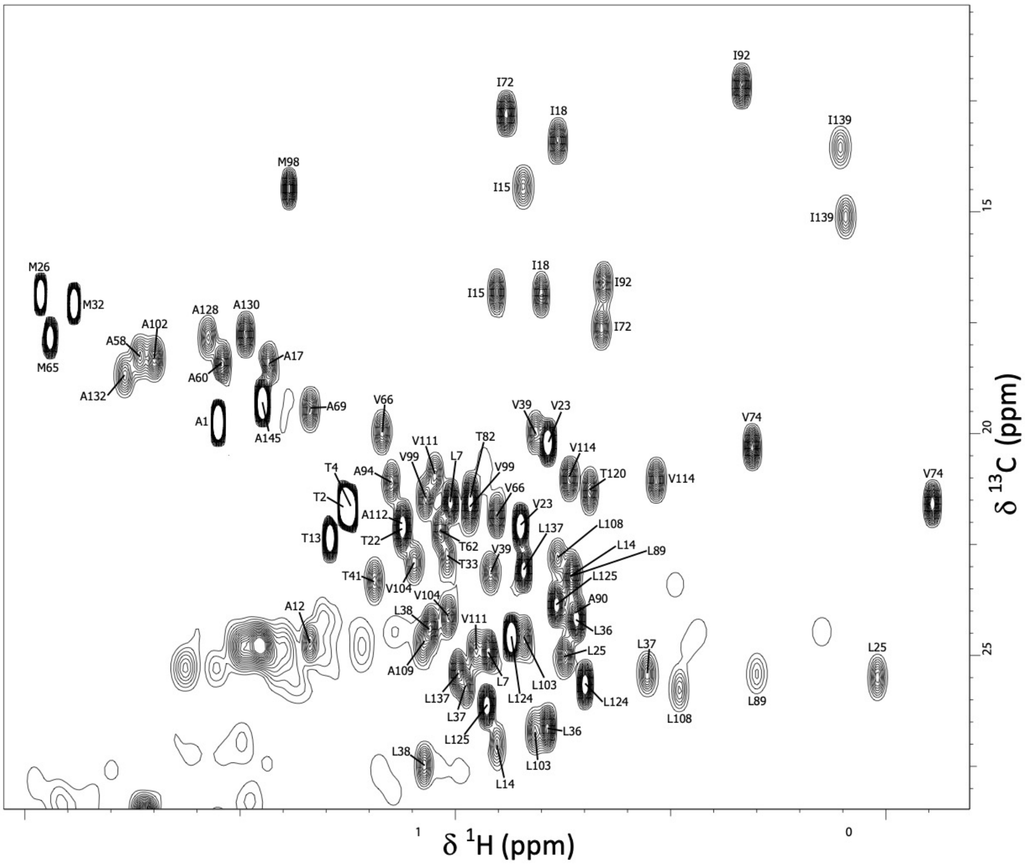

Figure S3: Assignment of Δ+PHS SNase CH_3 groups. [^{1}H-^{13}C] HSQC spectrum (zoom on the CH_3 cross-peaks region) of Δ+PHS SNase (same experiment as in Figure S2). CH_3 cross-peaks assignment is indicated using the one-letter amino acid and sequence number code.

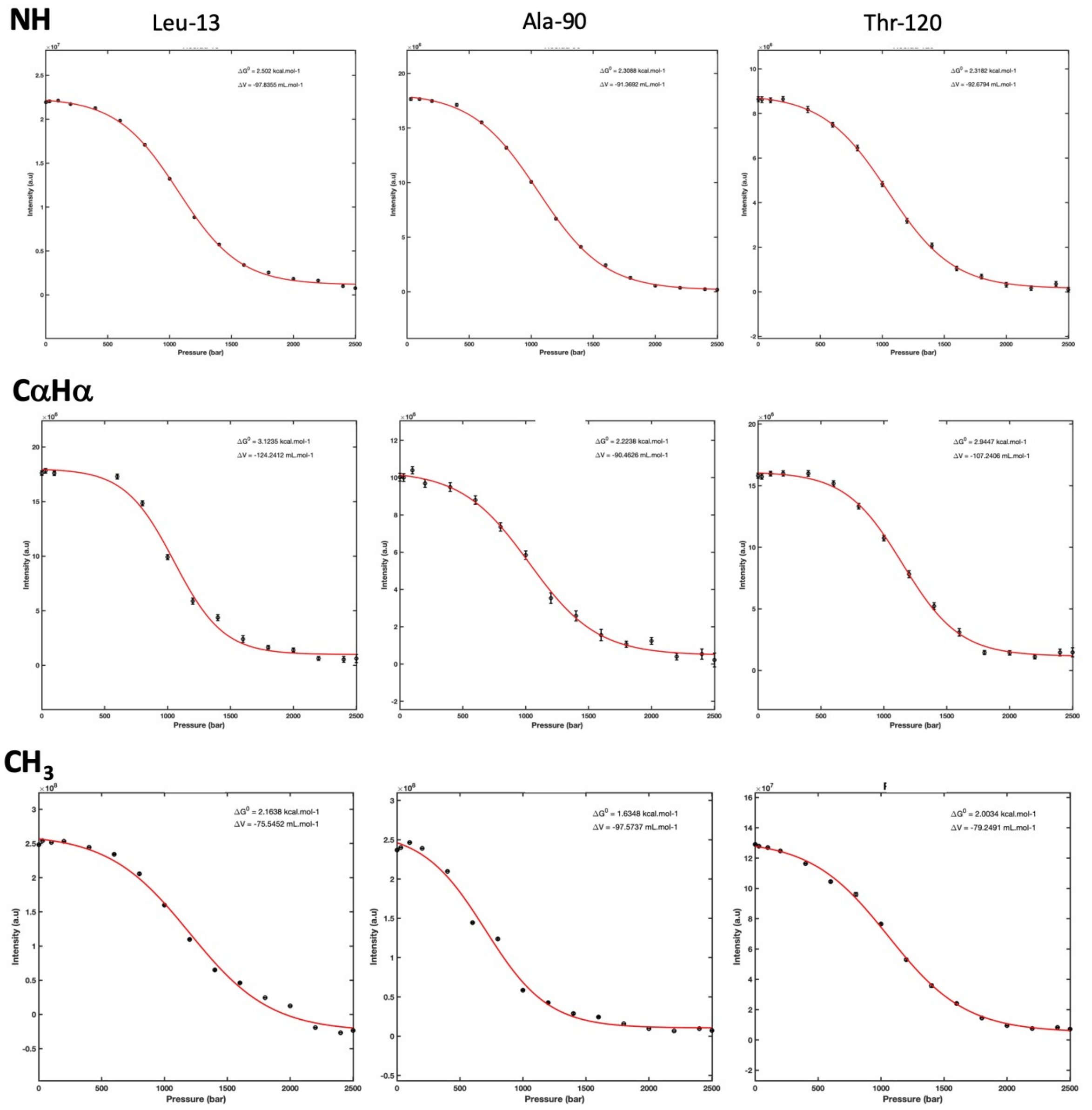

Figure S4: Representative examples of experimental fits obtained for the decrease in intensity with pressure of NH, CαHα, and CH_3 cross-peaks (from top to bottom) for the corresponding residues (L13, A90 and T120, as indicated on top of the graph) with Eq. [1] (Materials and Methods) implemented on an in-house MATLAB software. The complete collection of fits is available upon request to the corresponding authors.

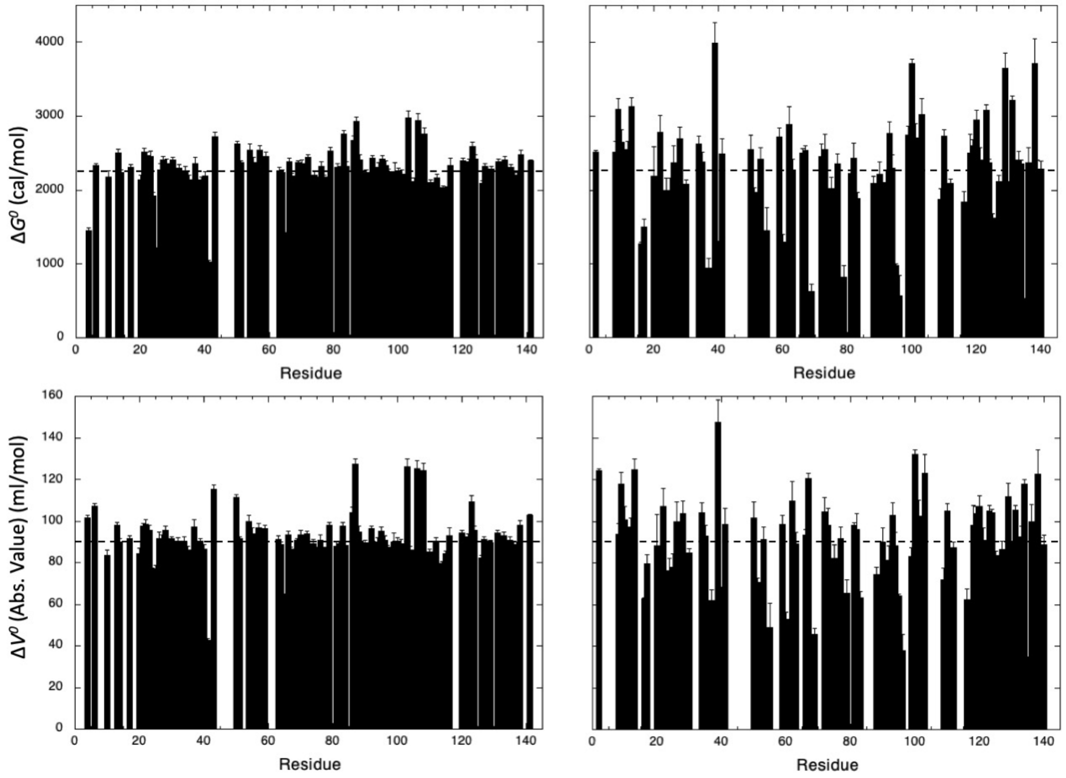

Figure S5. Thermodynamic parameters measured for the unfolding reaction of Δ+PHS SNase. ΔG^0 (upper panels) and ΔV^0 (absolute value, lower panels) obtained from the fit with Equation (1) of the pressure-dependent sigmoidal decrease of the residue cross-peak intensities of NH amide groups (left) and CαHα groups (right). The dashed lines represent the mean values of the measured thermodynamic parameters. Contrary to Figure 4, the values reported here concern all NH and CαHα groups for which accurate residue-specific denaturation curves has been obtained.

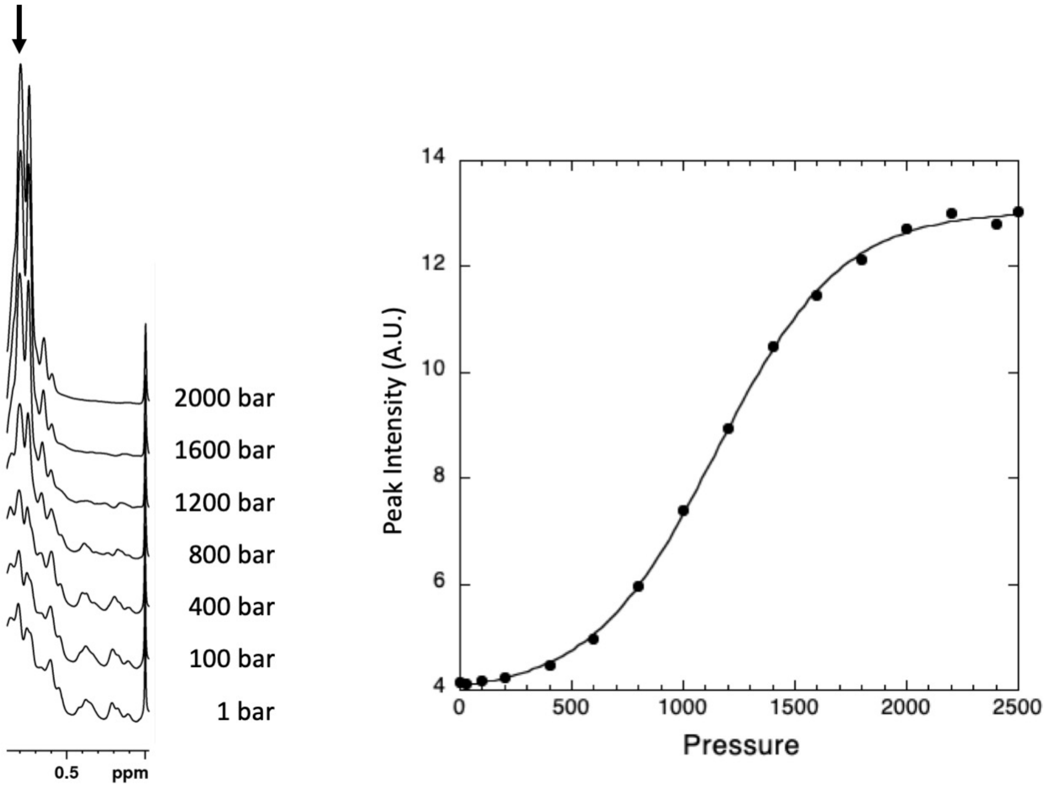

Figure S6. Monitoring the unfolding reaction of Δ+PHS SNase with 1D HP-NMR spectroscopy. Left: zoom on the methyl resonance region of the proton NMR spectra of Δ+PHS SNase recorded at increasing pressure. The arrow indicates the resonance (0.8 ppm) used to follow the unfolding reaction: it corresponds to the resonance of unfolded CH_3 that increases with pressure. Right: denaturation curve obtained from the fit of the evolution with pressure of the denatured methyl resonance of with a two states equilibrium equation (Equ.[1]).

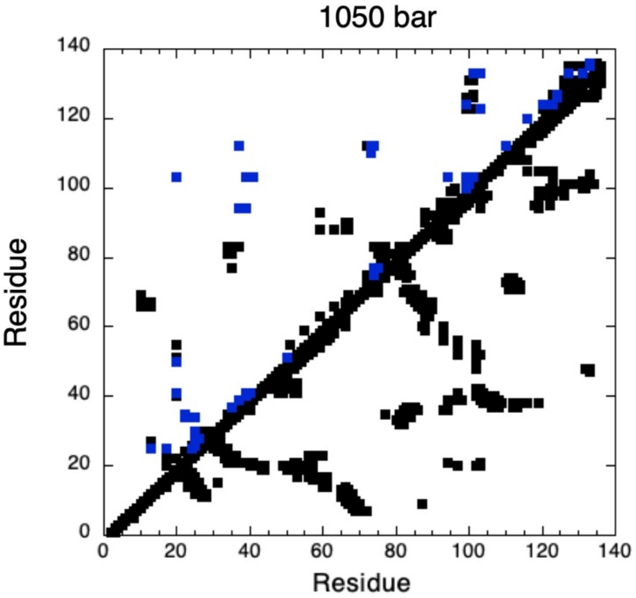

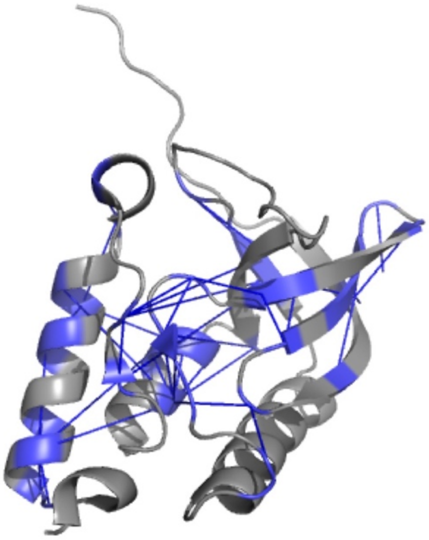

Figure S7. Fractional contact map obtained at 1050 bar from NH probes. Upper panel: contact maps built from the crystal structure of Δ+PHS Snase (PDB ID: 3LX0) at 1050 bar. Contacts below the diagonal have been calculated with CMview: they correspond to residue where the distance to the corresponding Cα is lower than 8.5 Å. Above the diagonal, the contacts displayed correspond to residues for which fractional probability can be measured from normalized residue-specific denaturation curves obtained from NH cross-peaks. In addition, contacts have been colored in blue when contact probabilities P_{ij} lower than 0.5 are observed. Lower panel: visualization of the probabilities of contact on ribbon representations of Δ+PHS SNase at 1050 bar. The blue lines represent contacts that are significantly weakened ($P_{ij} \leq 0.5$) at the indicated pressure. Residues involved in these contacts are also colored in blue.
